# Role of ceramide synthase 2 in G-CSF signaling and G-CSF-R translocation into detergent-resistant membranes

**DOI:** 10.1038/s41598-018-37342-8

**Published:** 2019-01-24

**Authors:** Jennifer Kurz, Julia Barthelmes, Leonard Blum, Thomas Ulshöfer, Marthe-Susanna Wegner, Nerea Ferreirós, Luise Roser, Gerd Geisslinger, Sabine Grösch, Susanne Schiffmann

**Affiliations:** 1 Fraunhofer Institute for Molecular Biology and Applied Ecology IME, Branch for Translational Medicine and Pharmacology TMP, Theodor-Stern-Kai 7, 60596 Frankfurt am Main, Germany; 2 pharmazentrum frankfurt/ZAFES, Institute of Clinical Pharmacology, Goethe University Hospital Frankfurt, Theodor-Stern-Kai 7, 60590 Frankfurt/Main, Germany

## Abstract

Ceramides are sphingolipids with defined acyl chain lengths, which are produced by corresponding ceramide synthases (CerS1-6). In experimental autoimmune encephalomyelitis (EAE), an animal model of multiple sclerosis (MS), the ablation of CerS2 suppresses EAE-pathology by reducing neutrophil migration into the central nervous system. This migration is induced by granulocyte-colony stimulating factor (G-CSF) signaling. G-CSF signaling leads to a signal cascade including the phosphorylation of Lyn kinase and STAT3. This in turn regulates expression of the neutrophil surface receptor chemokine receptor 2 (CXCR2) and causes translocation of the receptor into detergent-resistant membranes (DRMs). In this study we investigated the role of ceramides in G-CSF signaling. We found, that G-CSF treatment of wild type bone marrow cells (BMCs) leads to translocation of G-CSF-receptor (G-CSF-R) into DRMs. G-CSF also induces downregulation of ceramides in WT and CerS2 null BMCs, as well as upregulation of very long chain lactosylceramides. However, in CerS2 null BMCs, G-CSF failed to induce translocation of G-CSF-R into DRMs, leading to reduced phosphorylation of Lyn and reduced CXCR2 expression. Interestingly, G-CSF signaling in CerS6 null BMCs was not affected. In conclusion, very long chain ceramides are important for G-CSF signaling and translocation of G-CSF-R into DRMs.

## Introduction

Multiple sclerosis (MS) is a neurodegenerative autoimmune disease characterized by the infiltration of immune cells into the central nervous system (CNS). The infiltrating immune cells release chemokines to recruit further immune cells and cytokines/inflammatory mediators to promote the inflammatory process, leading to the death of oligodendrocytes and subsequently to demyelination and neurodegeneration. A commonly used animal model for MS is experimental autoimmune encephalomyelitis (EAE), which has been used successfully for translation of drug candidates, including mitoxantrone, glatiramer acetate and natalizumab, into human therapeutic approaches^1^. Among other immune cells involved in the pathogenesis of EAE, neutrophils are the first to infiltrate into the CNS^2–4^. Migration into the CNS occurs after neutrophil expansion in the bone marrow and accumulation in the peripheral vascular system^3^.

Granulocyte colony-stimulating factor (G-CSF) induces migration of neutrophils by upregulation of the chemokine receptor CXCR2. The binding of G-CSF to its receptor (G-CSF-R) leads to the dimerization of the extracellular domain of the receptor that in turn activates Lyn kinase and Janus kinases (Jak). These kinases phosphorylate one or more tyrosine residues in the C-terminal region of the G-CSF-R leading to the activation of multiple intracellular signaling proteins, including the signal transducers and activators of transcription (STAT) and mitogen-activated protein (MAP) kinases^5–7^. This signaling pathway is also regulated by suppressor of cytokine signaling 3 (SOCS3) which is induced by STAT3 and inhibits the catalytic activity of Jak and thereby, the expression of STAT3. This results in negative feedback regulation^8,9^. Activated STAT3 translocates into the nucleus and induces the expression of several genes such as CXCR2^10^.

The G-CSF-R is expressed on mature neutrophils in bone marrow and blood^11^. Binding of the ligand G-CSF to the G-CSF-R activates neutrophils and promotes their adhesion to the endothelium and subsequently, the migration of the cells into tissues^12^. The CXCR2 receptor is the primary mediator of CXCL2 chemokine signaling^11^ and antagonism of CXCR2 ameliorates clinical symptoms in EAE mice^13^. Recently, we demonstrated that expression of CXCR2 in neutrophils induced by G-CSF is regulated by ceramide synthases (CerS2 and CerS6). Furthermore, ablation of CerS2 or CerS6 leads to an amelioration or worsening of EAE pathology, respectively, and is linked to CXCR2 expression and neutrophil infiltration^2,14^.

The six known ceramide synthases are expressed in a tissue-specific manner and synthesize ceramides (Cer) by catalyzing the N-acylation of a long chain base such as sphingosine to fatty acyl chains of specific lengths. Ceramides are sphingolipids, which have emerged as interesting targets for MS therapy since FTY720 (fingolimod), a sphingosine analogue, has been used successfully in MS treatment, acting to confine lymphocytes to the lymphoid organs^15^. CerS2 synthesizes predominantly C22-C24-ceramides (very long chain ceramides), whereas CerS6 produces mainly C16-ceramides (long chain ceramides)^16,17^. The ratio between long chain ceramides and very long chain ceramides may influence the fluidity of the membrane and therefore the activation potential of membrane receptors^18^.

Within the outer leaflet of the cell membrane bilayer complex sphingolipids such as glycosphingolipids and cholesterol cluster together in distinct domains, the so called lipid rafts, along with glycosyl-phosphatidylinositol (GPI)-anchored proteins^19^. For lipid raft characterization cell membranes can be divided into biochemical fractions, which are non-soluble in detergents such as Triton X-100. Therefore, these fractions are called detergent-resistant membranes (DRMs). DRM isolation is a useful tool to identify proteins, which are situated in lipid rafts microdomains^20^. Previously it was shown that the sphingolipid composition of the membrane can influence the localization of membrane proteins such as CD36 or connexin 32^21^. It is still unclear whether this is due to the lack or elevation of specific sphingolipids or the fluidity of the membrane.

Our previous results indicate that G-CSF regulates CerS2 and CerS6 expression, which in turn mediates CXCR2 expression^2,14^. The mechanisms by which G-CSF induces the expression of CerS2 and CerS6, and how CerS2 and CerS6 mediate the regulation of CXCR2 are currently unknown. In this study, we investigate the hypothesis that a deficiency in ceramides with specific acyl chain lengths alters membrane properties and thereby prevents or promotes G-CSF signaling.

## Results

### G-CSF and G-CSF-R levels are not regulated by CerS2 during the preclinical phase of EAE

**Figure 1:**
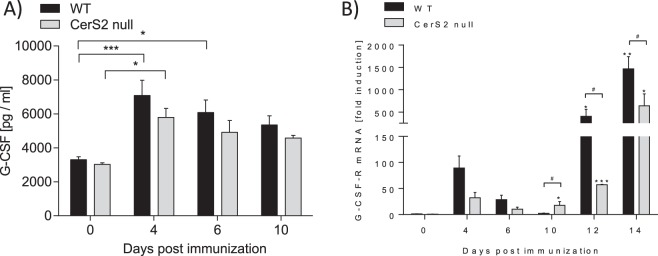
G-CSF protein and G-CSF-R mRNA levels are not regulated in a CerS2-dependent manner. (**A**) G-CSF protein levels in plasma and (**B**) G-CSF-R mRNA levels in white blood cells of CerS2 null and WT EAE mice at various disease time points including the preclinical phase (day 0-day 6) and acute disease phase (day 10-day 14). G-CSF protein levels were determined by ELISA. Pooled data are shown from three mice for each time point. The mRNA expression levels were normalized to peptidyl propyl isomerase A (PPIA) and were calculated using the mRNA level of untreated WT mice of the same age. Pooled data are shown from two (day 0/10/12/14) or three mice (day 4/6). The mRNA measurements were carried out in triplicate. Data are means ± SEM. *(p < 0.05) indicates significant differences within a group and ^#^(p < 0.05) between the two groups. Statistical analysis was performed by Two-way ANOVA.

Recently, we observed amelioration of EAE pathology in CerS2 null mice due to a reduced migration potential of neutrophils^2^. The impaired neutrophil migration was linked to reduced CXCR2 expression on neutrophils in the preclinical phase^2^. Because G-CSF induces CXCR2 expression, we suggested that CerS2 is required for proper activation of the G-CSF-R and thereby promotes the expression of CXCR2 and the neutrophil migration potential. To exclude the possibility that higher CXCR2 expression in EAE WT mice compared to EAE CerS2 null mice^2^ is due to increased basal levels of G-CSF or G-CSF-R, G-CSF levels in plasma and G-CSF-R mRNA levels in white blood cells (WBCs) of CerS2 null and WT EAE mice were measured. As expected, G-CSF levels increased in WT EAE mice at four days post immunization (Fig. 1A). However, the G-CSF levels were similar in CerS2 null and WT EAE mice during the preclinical phase and the onset of symptoms (day 0–day 10) (Fig. 1A). The expression of the G-CSF-R mRNA transiently increased in WBCs during the preclinical phase (day 0–day 6) (Fig. 1B). Also, G-CSF-R mRNA expression was similar in CerS2 null EAE mice and in WT EAE mice during the preclinical phase (Fig. 1B). After disease onset (day 10–day 14), G-CSF-R expression increased steadily in WBCs, whereas the G-CSF-R expression was significantly lower in CerS2 null cells compared to WT cells. However, these data indicate that neither the G-CSF nor the G-CSF-R levels are regulated by CerS2 in the preclinical phase of disease (day 0–day 6), which is critical for neutrophil migration into the CNS.

### G-CSF-R translocates into DRMs after activation

**Figure 2:**
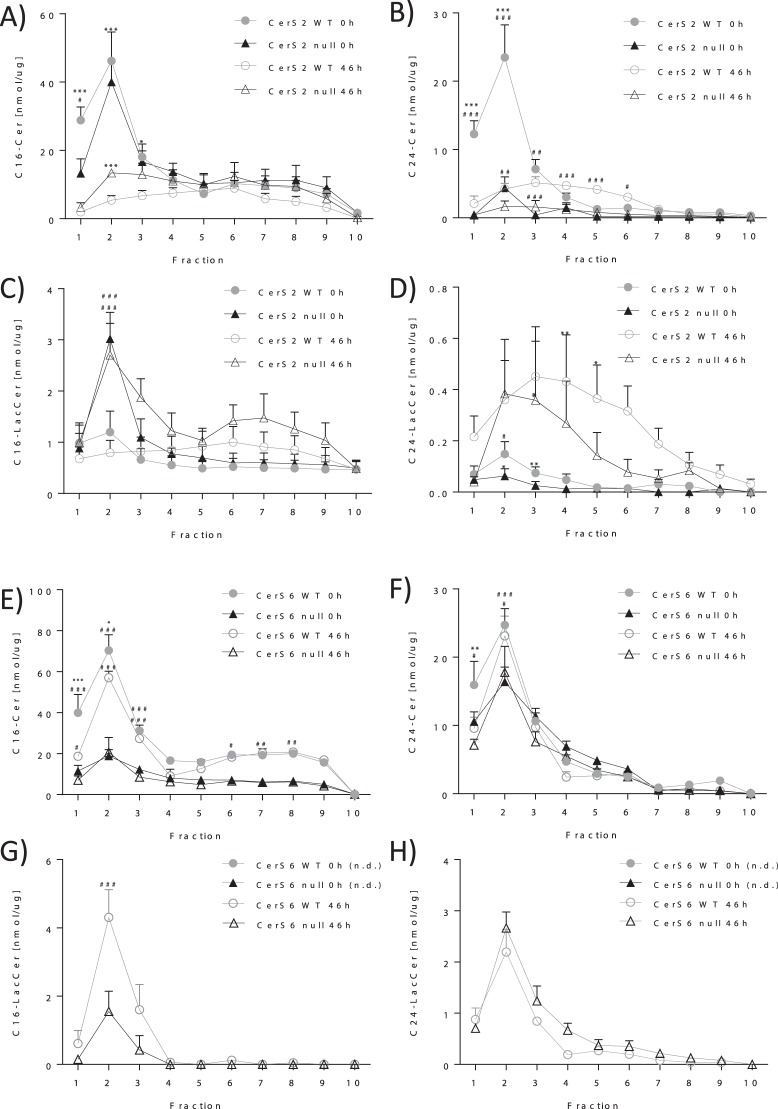
G-CSF alters ceramide composition in DRMs. C16-Cer, C24-Cer, C16-LacCer, C24-LacCer levels in the fractions of WT, CerS2 null (**A**–**D**) and CerS6 null (**E**–**H**) bone marrow cells (BMCs) before (0 h) and after 46 h treatment with G-CSF. BMCs were homogenized and fractionated on sucrose density gradients. Each fraction was analyzed for ceramide levels using LC-MS/MS. The figures show data from three (CerS2 WT/null BMCs; CerS6 WT/null 46 h; n = 6: CerS6 WT 0 h) to seven (CerS6 null 0 h) independent experiments each performed in duplicate. For one experiment, BMCs from two mice were pooled. Data are means ± SEM. *(p < 0.05) **(p < 0.01), ***(p < 0.001) indicates significant differences between time point 0 h and 46 h (Two-way ANOVA). ^#^(p < 0.05) ^##^(p < 0.01), ^###^(p < 0.001) indicates significant differences between WT and CerS2 null or CerS6 null cells at a specific time point (Two-way ANOVA). LC-MS/MS detection range: C16-Cer (200–20.000 pg), C24-Cer (2000–160.000 pg), C16-LacCer (2000–160.000 pg), C24-LacCer (200–20.000 pg), n.d. = not detectable.

**Figure 3:**
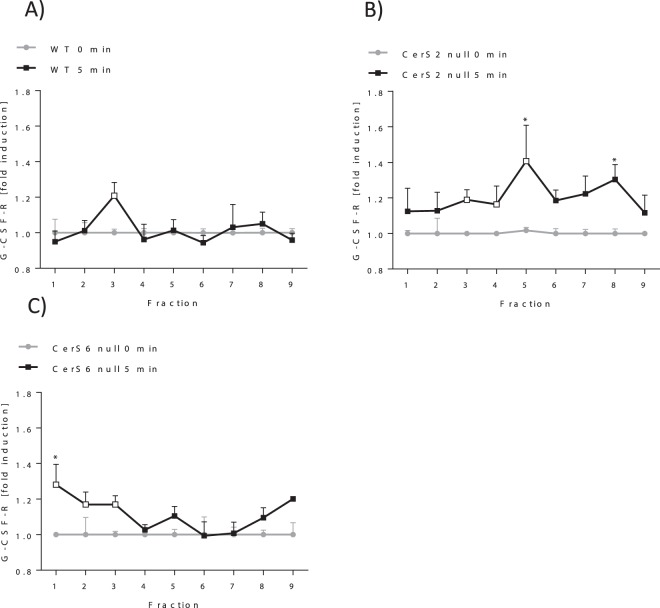
G-CSF induces translocation of G-CSF-R into DRMs. The relative G-CSF-R protein content in the fractions of G-CSF (5 min) treated bone marrow cells (BMCs) of WT (**A**), CerS2 null (**B**) and CerS6 null (**C**) mice. BMCs were homogenized and fractionated on sucrose density gradients. Each fraction was analyzed for the G-CSF-R protein levels using ELISA. The relative G-CSF-R levels were calculated using the protein level of unstimulated cells (0 h) as reference. The figure represents data from three experiments each achieved in duplicate. For one experiment, BMCs from two mice were pooled. Data are means ± SEM. *(p < 0.05), **(p < 0.01) and ***(p < 0.001) indicate significant differences between time point 0 h and 5 min within a group analyzed by t-test. Empty data points mark highest G-CSF-R fold induction after 5 min G-CSF stimulation.

Some receptors such as the insulin receptor translocate into DRMs following activation. However, in CerS2 null hepatocytes, the insulin receptor is not able to translocate into the DRMs. This in turn impairs its phosphorylation^21^. Therefore, we investigated whether the activation of the G-CSF-R by G-CSF induces translocation into DRMs and whether the translocation depends on the presence of very long chain ceramides produced by CerS2. Because the lack of CerS6 increases the expression of the G-CSF-R and worsens EAE pathology^14^, we also investigated whether the activation of the G-CSF-R is CerS6 dependent. First, we identified the fractions that contain the DRMs in CerS2/6 null and WT bone marrow cells (BMCs) by determination of sphingolipid and cholesterol content. The DRMs from BMCs were isolated using sucrose gradient centrifugation. The DRMs were located predominantly in fraction two, as determined by the cholesterol content in BMCs from WT mice (Supplemental 2A,B). This was confirmed by the finding that sphingolipids such as C16-Cer, C18-Cer, C20-Cer and C24-Cer were also located mainly in fraction two (Fig. 2A,B,E,F (0 h), Supplemental 3A–D, Supplemental 4) of CerS2 WT DRMs without G-CSF stimulation (0 h). In CerS2 null cells, the DRMs were also predominantly located in fraction two, as shown by a significantly higher level of C16-Cer, C18-Cer, C20-Cer and LacCer16 in fraction 2 in comparison to fraction 4 (Fig. 2A,C (0 h), Supplementals 3 and 4). In CerS6 null BMCs, cholesterol was mainly located in fraction 1–3 (Supplemental 2A,B), whereas ceramide concentrations were highest in fraction 2 (Fig. 2E–H (0 h), Supplemental 3C,D). To investigate whether the G-CSF-R translocates into the DRMs after activation by G-CSF, BMCs were incubated with G-CSF for 5 min and the protein levels of G-CSF-R were determined in each fraction by ELISA. The G-CSF-R protein levels were increased in fraction three in WT BMCs after activation by G-CSF, indicating the translocation of the G-CSF receptor into the DRM fraction (Fig. 3A). Interestingly, the G-CSF-R protein levels were increased in nearly all fractions in CerS2 null BMCs (Fig. 3B) and in fractions 1–3 in CerS6 null cells (Fig. 3C). These data imply that the G-CSF-R translocates into the DRM in WT and CerS6 null BMCs but not in CerS2 null cells.

### Phosphorylation of Lyn kinase is regulated in a CerS2 dependent manner

**Figure 4:**
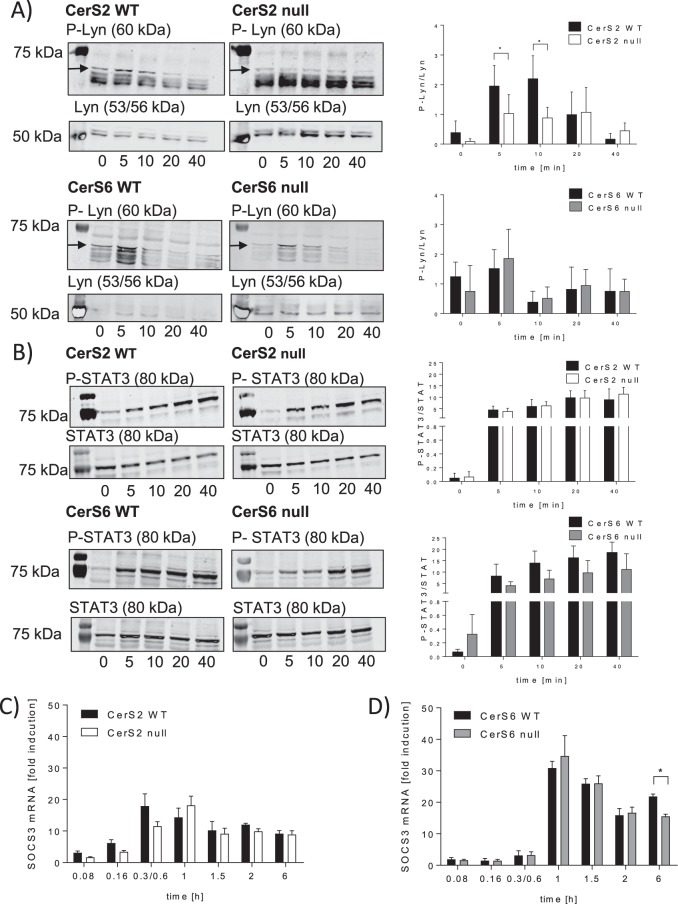
Downstream signaling of G-CSF is regulated in a CerS2-dependent manner. (**A**) Western blot and densitometric analysis of P-Lyn kinase (60 kDa) and Lyn kinase (53/56 kDa) (**B**) P-STAT3 (80 kDa) and STAT3 (80 kDa). Bone marrow cells (BMCs) from WT, CerS2 null and CerS6 null mice were incubated for 5, 10, 20 and 40 min with 10 ng/ml G-CSF and the phosphorylation of Lyn kinase and STAT3 was determined using western blot. The relative phosphorylation status of the kinases and the transcription factor were calculated using the unphosphorylated protein as reference. (**C**,**D**) Time dependent SOCS3 mRNA expression in WT, CerS2 null or CerS6 null cells stimulated with G-CSF. The mRNA expression levels were normalized to peptidyl propyl isomerase A (PPIA) and were calculated using the mRNA level of untreated cells at time point 0 h. A representative western blot of three is shown. Blots are cropped to the areas of interest. Phosphorylated Lyn/STAT3 and unphosphorylated protein were detected on the same blots using different secondary antibodies. Full-length blots are enclosed in Supplemental 5. The densitometric analysis represents the data from three independent experiments. For one experiment, BMCs from two mice were pooled. The mRNA experiments were repeated at least six times and were done in triplicate. Data are means ± SD (**A**,**B**) and ± SEM (**C**,**D**). *(p < 0.05), **(p < 0.01) and ***(p < 0.001) indicate significant differences between WT cells and CerS2 null or CerS6 null cells analyzed by t-test.

We investigated whether the activation of the G-CSF-R is dependent on CerS2/6. The activation of the G-CSF-R leads to phosphorylation of the Lyn kinase and finally to STAT3 activation^22^. Therefore, we determined the phosphorylation status of the Lyn kinase and STAT3 in CerS2/6 null and WT BMCs upon G-CSF stimulation. G-CSF led to a transient phosphorylation of the Lyn kinase in WT and CerS6 null BMCs, whereas in CerS2 null BMCs the phosphorylation of the Lyn kinase was not induced by G-CSF (Fig. 4A). Furthermore, G-CSF led to a phosphorylation of STAT3 in WT BMCs. Unexpectedly, in CerS2 null BMCs, the phosphorylation of STAT3 was not altered, whereas in CerS6 null BMCs the phosphorylation of STAT3 was slightly, but not significantly reduced, compared to WT cells (Fig. 4B). These data indicate that the phosphorylation of Lyn is CerS2-dependent, whereas STAT3 seems to be independent of regulation by CerS2/6.

### SOCS3 expression is not CerS2 or CerS6 dependently regulated

Next we investigated, whether the suppressor of cytokine signaling 3 (SOCS3) is CerS2 or CerS6 dependently regulated. SOCS3 is a key negative regulator for G-CSF signaling^23^ which binds to the catalytic domain of Jak2^24^. SOCS3 mRNA expression was measured after time dependent G-CSF treatment in CerS2/6 WT and null BMCs. A transient increase of SOCS3 mRNA was detectable in WT, CerS2 null and CerS6 null BMCs after G-CSF stimulation. However, in CerS2 null BMCs, SOCS3 mRNA was increased with a slight time shift in comparison to WT BMCs (Fig. 4C). CerS6 WT and CerS6 null BMCs exhibited a comparable transient increase in SOCS3 (Fig. 4D).

### Expression of CXCR2 is regulated by CerS2

**Figure 5:**
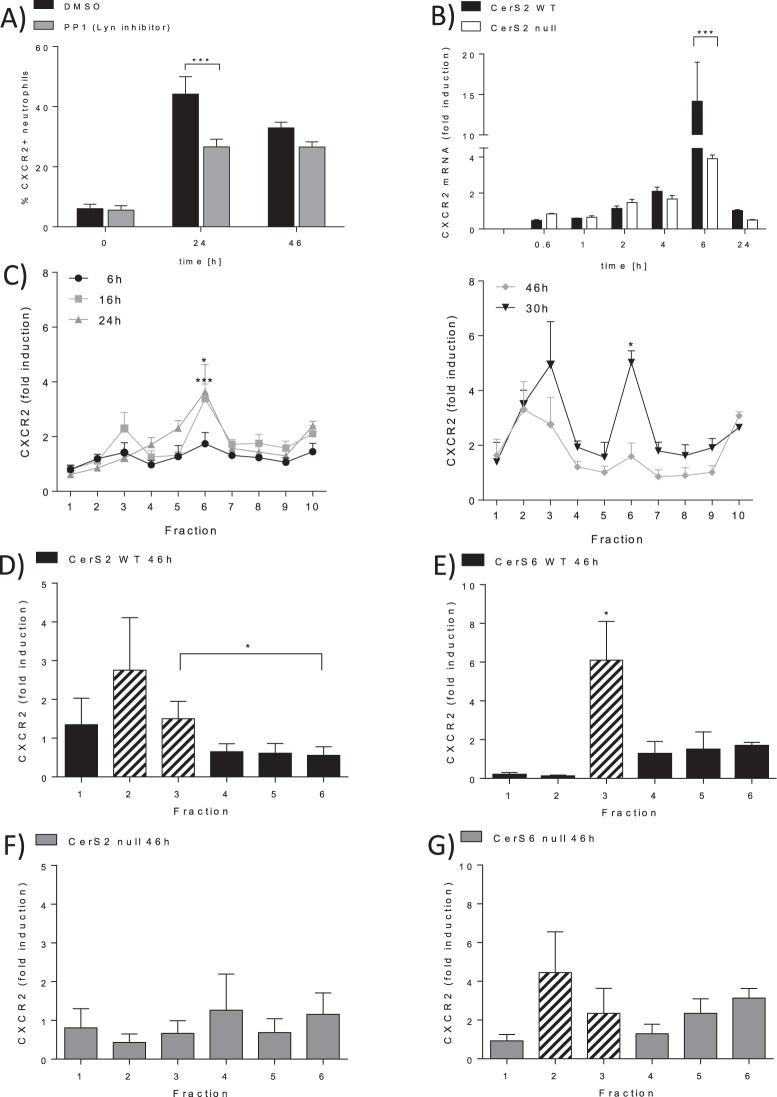
CXCR2 expression is regulated in a CerS2 dependent manner. (**A**) CXCR2 protein expression is induced by G-CSF via Lyn signaling. WT bone marrow cells (BMCs) were treated with 10 ng/ml G-CSF at the indicated time points in the presence of 10 µM PP1 (Lyn inhibitor) or DMSO (control). CXCR2 expression was determined using flow cytometry. The figure shows the percentage of CXCR2 positive neutrophils from 2–3 independent experiments (**B**) Relative CXCR2 mRNA expression in BMCs isolated from WT or CerS2 null treated with 10 ng/ml G-CSF for the indicated time points. The mRNA expression levels were normalized to peptidyl propyl isomerase A (PPIA) and were calculated using the mRNA level of untreated cells as reference. The figure represents the data from six independent experiments, each achieved in triplicate. (**C**) Time dependent relative CXCR2 protein expression in WT BMCs stimulated with 10 ng/ml G-CSF. CXCR2 protein levels were determined by ELISA. Time point 0 h was used as protein level reference. The figure shows data from two independent experiments, each carried out in duplicate. (**D**,**G**) CXCR2 protein expression in WT (**D**,**E**), CerS2 null (**F**) and CerS6 null (**G**) cells. BMCs were homogenized and fractionated on sucrose density gradients. Each fraction was analyzed for CXCR2 protein levels using ELISA. The relative CXCR2 levels were calculated using the protein level of untreated cells as reference. Figures show data from four (CerS2) and three (CerS6) independent experiments, each experiment was performed in duplicate. Data are means ± SEM. Dashed bars indicate DRM fractions. *(p < 0.05), **(p < 0.01) and ***(p < 0.001) indicate significant differences between DMSO-treated and PP1 treated WT cells (**D**,**E**), between WT cells and CerS2 null (**F**) or CerS6 null cells (**G**) or differences between time points in C57BL/6 DRMs analyzed by Two-way ANOVA (**A**–**C**) and t-test (**D**–**G**).

It is known that G-CSF stimulates the expression of CXCR2 via STAT3 in neutrophils^10^. However, to our knowledge it is unknown if Lyn kinase signaling plays a role in CXCR2 expression in neutrophils. Therefore, we investigated whether Lyn kinase also influences CXCR2 expression. We also checked whether an inhibitor of the Lyn kinase (PP1) can prevent the upregulation of CXCR2 on neutrophils isolated from BMCs. The cells were treated with G-CSF for 24 h or 46 h in the presence or absence of the inhibitor. G-CSF induced upregulation of CXCR2, whereas PP1 prevents the increase of CXCR2 expression after 24 h (Fig. 5A). To exclude that the observed effect is due to apoptosis induction, we investigated whether PP1 induces apoptosis in BMCs. However, PP1 did not increase apoptosis rate in BMCs (Supplemental 3E). These data indicate that CXCR2 expression is regulated via Lyn signaling.

As our results demonstrate, ablation of CerS2 prevents phosphorylation of Lyn kinase after stimulation with G-CSF. Thus, we expected that the CXCR2 expression would be reduced in CerS2 null BMCs. As expected, G-CSF induced a time dependent expression of CXCR2 mRNA in WT BMCs, predominantly after 6 h (Fig. 5B). In CerS2 null BMCs, G-CSF-induced CXCR2 mRNA expression was significantly reduced at this time point (Fig. 5B). Protein expression was significantly reduced after 24 h and 46 h, a finding we observed previously^2^.

### Translocation of CXCR2 is CerS2 dependently regulated

Since CXCR2 is a membrane receptor, we examined whether CXCR2 translocates into the DRMs and whether this depends on ceramide status. BMCs isolated from C57BL/6 mice were treated with G-CSF for different time periods (6 h, 16 h, 24 h, 30 h and 46 h). In unstimulated BMCs, the CXCR2 was primarily localized in fraction 10, whereas the activation with G-CSF led to an increase of CXCR2 in fraction 6 after 24 h. This was followed by an increase of CXCR2 in the next 22 h, in fractions 2 and 3, accompanied by a decrease in CXCR2 in fraction 6, indicating that G-CSF induces the expression of CXCR2 and leads to its translocation into DRM fractions 2 and 3 (Fig. 5C). Therefore, CerS2/6 WT and null BMCs were investigated upon CXCR2 translocation into the DRMs after 46 h of G-CSF stimulation. After stimulation, WT BMCs exhibited an increased level of CXCR2 in DRM fractions two and three (Fig. 5D,E). In G-CSF activated CerS2 null cells, CXCR2 was not increased (Fig. 5F), whereas CXCR2 in G-CSF activated CerS6 null cells was localized in fraction two and three (Fig. 5G). These data indicate that G-CSF induced expression and translocation of CXCR2 is regulated in a CerS2 dependent manner, whereas CerS6 seems to have no effect on CXCR2 expression and translocation into DRMs.

### G-CSF induces alteration of DRM composition

Recently, we observed that G-CSF treatment induces CerS2 and CerS6 expression^2,14^. Therefore, we investigated whether this translates into an altered ceramide or cholesterol content of the DRMs. After 46 h G-CSF treatment cholesterol was found in fraction 2 or 3 in WT and CerS6 null cells, whereas in CerS2 null cells cholesterol was found in each fraction (Supplemental 2A,B). Surprisingly, C16-Cer and C24-Cer were reduced and C24-lactosylceramide (LacCer) was increased in DRMs isolated from G-CSF stimulated CerS2 WT BMCs after 46 h (Fig. 2A–D). In DRMs isolated from CerS2 null cells, C16-Cer was reduced. In addition C24-LacCer and C16-LacCer were increased after G-CSF stimulation (Fig. 2A–D (46 h)). In CerS6 WT and CerS6 null BMCs, C16-Cer and C24-Cer content remained unchanged (Fig. 2E,F (46 h)), whereas both lactosylceramides C16-LacCer and C24-LacCer increased after G-CSF stimulation (Fig. 2G,H (46 h)). In fractions isolated from CerS6 null cells ceramides were also mainly located in fraction 2 (Fig. 2E–H (46 h)). These data indicate that in DRMs the complex sphingolipids with a very long chain ceramide backbone might be important for the translocation of the G-CSF-R and CXCR2. This was demonstrated by the observation that CerS2 null cells lacking these sphingolipids are characterized by a loss of DRMs and of impaired G-CSF-R and CXCR2 translocation (Fig. 3 and 5D–G).

### G-CSF regulates CerS2 expression transcriptionally via Lyn

**Figure 6:**
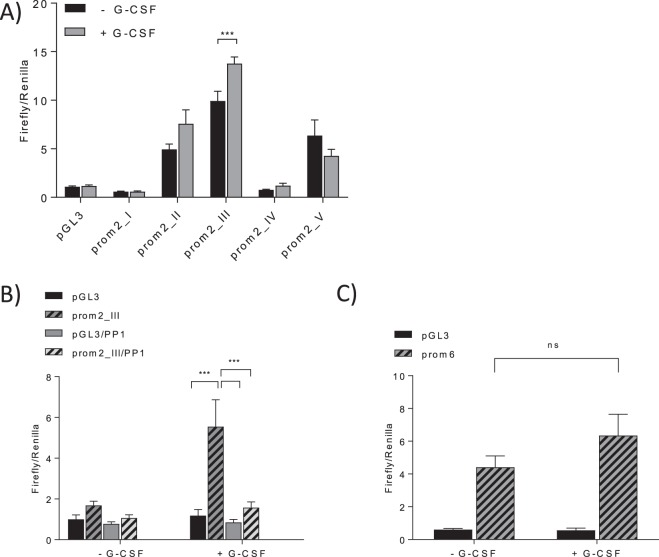
CerS2 mRNA expression is transcriptionally regulated by G-CSF. (**A**) Luciferase activity assay of CerS2 promoter reporter gene constructs in RAW247 cells after G-CSF treatment. RAW247 cells were transiently transfected with the CerS2 promoter luciferase constructs as well as with the pGL3 Basic vector (control vector without promoter). Twenty-four hours later they were treated with 10 ng/ml G-CSF for 46 h. (**B**) RAW247 were transiently transfected with the Prom2_III as well as the pGL3 Basic vector and treated 24 h later with 10 ng/ml G-CSF and 2.5 µM PP1 for 46 h. (**C**) RAW247 were transiently transfected with Prom6 (CerS6 promotor) as well as the pGL3 Basic vector and treated 24 h later with 10 ng/ml G-CSF for 46 h. Luciferase activity was normalized to the RLU of renilla luciferase (transfection control). The figures show the results from at least three independent experiments each carried out in quadruplicates. Data are means ± SEM. ns = not significant, **(p < 0.01) and ***(p < 0.001) indicate significant differences between DMSO-treated and PP1 treated RAW247 cells or between G-CSF-treated and vehicle-treated RAW247 cells analyzed by Two-way ANOVA.

To gain further insight into the transcriptional expression of CerS2 in response to G-CSF stimulation, a promoter luciferase reporter gene assay was performed. Five genomic DNA fragments, located upstream of the transcription start site of the murine CerS2 gene were cloned into the pGL3-plasmid (detailed promotor construct composition see Supplemental 1A). Transfection with the putative promoter constructs prom2_I/prom2_IV failed to induce luciferase activity in RAW247 cells (Fig. 6A). Prom2_II/prom2_V lead to moderate induction of luciferase activity. However, the transfection with the putative promotor prom2_III and subsequent stimulation of the cells with G-CSF resulted in the most distinct G-CSF-dependent induction of luciferase activity (Fig. 6A). These data indicate that the murine promotor of CerS2 is located on exon 1 and intron 1. Moreover, these data suggest that G-CSF inducible transcription factors also bind on exon 1 and/or intron 1.

Next, we sought to establish whether Lyn signaling contributes to the activation of the CerS2 promotor. Therefore, RAW247 cells were transfected with pGL3-prom2_III and subsequently treated for 46 h with G-CSF in the presence or absence of PP1. Interestingly, PP1 prevented the G-CSF induced luciferase activity indicating that Lyn kinase signaling is involved in the induction of CerS2 expression (Fig. 6B). To exclude the possibility that reduced cell viability was responsible for the reduced luciferase activity, the viability of transfected cells treated with PP1 was assessed. PP1 only slightly reduced cell viability after 46 h and is therefore unlikely to be responsible for the observed reduction of CerS2 expression (Supplemental 3F).

### G-CSF did not regulate CerS6 expression transcriptionally

A genomic DNA fragment (prom6), located upstream of the start codon of the murine CerS6 (Supplemental 1B) gene, was cloned into a pGL3 vector. Transfection with the putative CerS6 promotor prom6 increased luciferase activity in RAW247 cells indicating that the DNA fragment represents at least a part of the CerS6 promotor (Fig. 6C). However, G-CSF was not able to further increase the luciferase activity in prom6 transfected RAW247 cells. These data indicate that G-CSF did not induce CerS6 expression transcriptionally (Fig. 6C).

## Discussion

**Figure 7:**
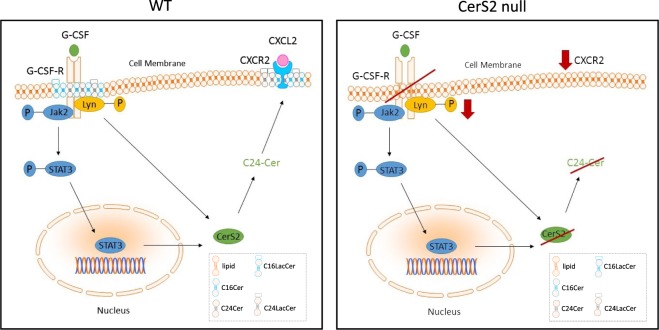
Postulated mechanism for ceramide dependent regulation of G-CSF signaling in BMCs. In WT cells, G-CSF leads to the translocation and activation of G-CSF-R followed by the phosphorylation of Lyn kinase and of STAT3. This ultimately results in the expression of CXCR2 and its translocation into DRMs. Furthermore, G-CSF leads to downregulation of ceramides (CerS2 WT) and to upregulation of glycosylated very long chain ceramides in the plasma membrane. In CerS2 null cells, G-CSF did not induce the translocation of the G-CSF-R or the phosphorylation of Lyn, while the expression of CXCR2 was impaired. Moreover, due to the lack of CerS2, G-CSF induces upregulation of glycosylated long chain ceramides, leading to an altered lipid composition of the plasma membrane of CerS2 null cells in comparison to WT cells. In CerS6 null cells, G-CSF signaling and membrane composition is comparable to that in WT cells. The diagram of the displayed pathway was created using Biomedical-PPT-Toolkit-Suite (motifolio.com).

Ceramides have been shown not only to provide structural integrity to cell membranes but to play an important role as second messengers in cell signaling. They are involved in crucial physiological processes such as cell differentiation, growth and apoptosis. Ceramides also regulate cell signaling processes in an acyl chain dependent manner. Our data revealed that the presence of very long chain ceramides is essential for G-CSF signaling. In detail, the lack of very long chain ceramides prevents G-CSF-induced translocation of the G-CSF-R into DRMs, phosphorylation of Lyn kinase and CXCR2 expression leading to reduced potential for neutrophil migration (Fig. 7). Accordingly, transcriptional CerS2 expression was induced by G-CSF. In contrast, long chain ceramides do not seem to influence G-CSF signaling in our study. However, we are unable to absolutely exclude the involvement of long chain ceramides in this study, since C16 ceramides have been shown to be increased in CerS2 null mice^18^.

Lipid rafts are defined as microdomains within the lipid bilayer of cellular membranes that assemble proteins and lipids (cholesterol and sphingolipids such as ceramides) and experimentally resist extraction in cold detergent (detergent-resistant membrane)^25^. In WT BMCs, DRMs consist of a mixture of ceramides and ceramides with sugar side chains (i. e. LacCer) of different chain lengths. In CerS2 null BMCs, DRMs also consist of ceramides and glucosylceramides but with almost no contribution of sphingolipids with a C24- and C24:1-Cer backbone. The lack of very long chain ceramides does not prevent the formation of DRMs but alters the composition of the DRM and the fraction location (at least in hepatocytes^21^) and thereby the biophysical properties of the membrane. The lack of very long chain ceramides in CerS2 null cells leads to a more fluid^26^ membrane and might influence cell signaling. For example the incorporation of 2-hydroxylated fatty acids (2OHFAs) into the cell membrane results in decreased lipid order and dipole potential as well as less rigid packing of acyl chains and hence, modulates membrane proteins by microdomain reorganization and membrane fluidity^27^. Furthermore, activation with G-CSF and thereby the switch to neutrophils leads to an increase in C24-LacCer in WT cells, whereas in CerS2 null cells, C16-LacCer and C24-LacCer were increased. This unexpected C24-LacCer increase in CerS2 null BMCs might be produced by CerS3 or CerS4, which are also capable of very long chain ceramide synthesis^17,28^. Also, in the dextran sodium salt (DSS) evoked model of ulcerative colitis, an increase in very long chain ceramides in CerS2 null mice was shown, accompanied by an increase in CerS3 mRNA expression^29^. The observed decrease in ceramides after G-CSF induction in CerS2 WT and null BMCs is possibly due to the fact that ceramides are precursors for the glucosylceramides. Additionally, the G-CSF induced altered membrane composition prevented the formation of DRMs in CerS2 null cells, as indicated by the lack of cholesterol accumulation in DRM-fractions (Supplemental 2A,B). However, this is in contrast to findings from Park and co-workers, who observed a shift of DRMs from fraction 1–3 in WT hepatocytes to fraction 4–6 in CerS2 null hepatocytes^21^.

The altered sphingolipid composition, the more fluid membrane and the lack of DRM in G-CSF-activated cells are possibly responsible for the lack of G-CSF-R translocation and the impaired G-CSF signaling, such as reduced Lyn phosphorylation and reduced CXCR2 expression. The importance of sphingolipid composition and altered membrane properties for the function of membrane receptors has already been observed by others. CD36 which is responsible for the uptake of triglycerides and the insulin receptor did not translocate into DRMs in CerS2 null hepatocytes and consequently loose their functions^21,30^. The gap junction protein connexin 32 displays a mislocalization in CerS2 null hepatocytes due to disruption of DRMs^31^. For FcγRIIB, it was shown that exclusion from DRMs prevents interaction with signaling molecules^32,33^ and therefore, possibly their inhibitory potential in immune regulation. In CerS2 null hepatocytes, TNFR1 is not internalized and inhibits selective pro-apoptotic downstream signaling for apoptosis^34^.

We cannot exclude that ceramides synthesized by sphingomyelinase (SMase) also contribute to the observed effects, since ceramides generated by SMase enhance Tyr-phosphorylation of Jak2 and subsequently STAT3 phosphorylation in fibroblasts^35^. The existence of cholesterol and sphingolipid rich microdomains is a prerequisite for this activation. It is also possible that ceramides directly interact with proteins and thereby, induce dephosphorylation of Jak2 or Lyn kinase. For example, C18 ceramide activates protein phosphatase-2A^36^ which in turn, can dephosphorylate Jak2^37^.

As it has already been shown by others, our data confirm that G-CSF induces the Lyn signaling cascade and leads to activation of STAT3^38,39^. G-CSF also induced CerS2 promotor activity. To identify which transcription factor, activated by G-CSF signaling, was responsible for CerS2 promotor activity, we analyzed five different upstream regions of the start codon of CerS2. Promotor construct prom2_III was able to function as a promotor. Prom2_III comprises exon 1 and intron 1. To narrow down the putative binding site, we took into account that construct prom2_IV lacks the first 643 Bp of intron 1 and has no promotor activity. These data suggest that the transcription factor binds to intron 1 at the first 643 Bp and/or to exon 1. This is in line with the findings of Gong *et al*. who identified exon 1 as a regulatory region for gene transcription in human CerS2 and as binding site of the transcription factor SP1, which interacts with KLF6^40^. Analysis of the putative promotor region DNA fragment of CerS2 including exon 1 and 700 Bp from intron 1 by the “Promo program” (maximum matrix dissimilarity rate: 5%)^41,42^ suggests several putative transcription factor binding sites. This includes CCAAT/enhancer-binding protein C/EBPα, C/EBPβ, IRF-1 and STAT5 which are induced by G-CSF or belong to G-CSF-responsive genes^22^, but no STAT3 binding site. These data indicate that CerS2 mRNA expression is not induced via STAT3 but via a yet unknown transcription factor.

Previously, we demonstrated that the genetic deletion of CerS2 ameliorates clinical symptoms in EAE mice. We showed that the ameliorated clinical symptoms in CerS2 null mice were due to a reduced potential for migration of neutrophils that express reduced CXCR2 protein levels. In this study, we showed that in CerS2 null cells, translocation of the G-CSF-R is prevented leading to impaired receptor function and subsequently, to reduced expression of CXCR2. We assume that besides the G-CSF-R, other DRM-located proteins that regulate the immune response or inflammatory processes can be influenced by the alteration of DRM properties in CerS2 null mice. Further insight into the influence of chain-length specific ceramides on cell signaling is therefore important to better understand their involvement in diseases such as cancer and autoimmune diseases and to pinpoint possible therapeutic targets.

## Material and Methods

### Cells and reagents

BMCs were cultured and stimulated in RPMI 1640 GlutaMAX medium containing 2.5% hormone-free fetal calf serum (FCS) and 1% penicillin/streptomycin. RAW247 macrophages were cultured in RPMI 1640 medium – GlutaMAX containing 10% FCS. For the reporter gene assay, RAW247 macrophages were incubated in RPMI 1640 medium – GlutaMAX containing 2.5% FCS (charcoal stripped or normal). Cells were cultured at 37 °C in an atmosphere containing 5% CO_2_. G-CSF was purchased from BioLegend (Fell, Germany).

### Animal models

In all experiments, the ethics guidelines for investigations in conscious animals were followed and the experiments were approved by the local Ethics Committee for Animal Research (Regierungspräsidium Darmstadt, F152/03). Unless otherwise stated, mice for all experiments were female, between 8–14 weeks of age.

### EAE mouse model

10- to 13-week-old female 129S4/SvJae × C57BL/6 mice (F1 WT or F1 CerS2 null) were used for induction of EAE. The induction was achieved as recommended by the supplier using an EAE kit of Hooke Laboratories (Lawrence, USA). The induction of the EAE model has been described previously^2^. Untreated mice were used as the control group.

### Determination of G-CSF

The concentration of G-CSF in blood plasma was determined using a mouse G-CSF enzyme-linked immunosorbent assay (ELISA) kit (Abcam, Cambridge, UK) according to the manufacturer’s instructions. Blood plasma was isolated by centrifugation (300 g, 10 min), stored at −80 °C and diluted 1:50 in VE-H_2_O. The absorbance was determined using an Infinite 200 PRO Reader (Tecan, Crailsheim).

### Isolation of bone marrow cells

Animals were killed and their femurs and tibias removed aseptically. Bone marrow cavities were flushed with phosphate buffered saline (PBS) using a 27G1/2 needle. Single cell suspensions were prepared by repeated pipetting and passing through a 70 μm nylon filter. After centrifugation at 4 °C and 400 g for 3 min, cells were resuspended in 0.5 ml of erythrocyte lysing solution (150 mM NH_4_Cl, 100 mM NaHCO_3_, 0.1 mM Na-EDTA pH 7.4) and incubated for 5 min before adding 5 ml of cold PBS. Cells were centrifuged, washed with PBS, and counted using a hemocytometer. For the experiments BMCs from 2 mice were pooled.

### Isolation of detergent-resistant membranes

BMCs from two CerS2/6 null and two WT animals (usually 2.5–4.5 × 10^7^ cells/ml) were stimulated with 10 ng/ml G-CSF for 5 min or 46 h in RPMI 1640 GlutaMAX medium. 5 min G-CSF stimulation was performed for determination of G-CSF-R levels by ELISA, since the activation of the receptor occurs at the beginning of the signaling cascade. Cellular processes with longer time spans, such as CXCR2 activation/translocation and production of ceramides were measured after 46 h. Cells were scraped off, washed with PBS and stored at −20 °C. 67.5%, 35% and 5% sucrose was freshly prepared and dissolved in water. The BMCs were thawed on ice and resuspended in 566 μl MES-buffer (25 mM MES, 0.15 M NaCl, pH 6.5, 1% Triton X-100 and 1 x proteinase-inhibitor (Complete Mini, Roche Diagnostic, Mannheim)). Cells were homogenized and sonicated 5 times for 20 s at level 3 with constant ultrasonic frequency (Branson Sonifier 250, Schwäbisch Gmünd). BMCs were kept on ice and resolved thoroughly in 1.13 ml 67.5% sucrose and filled into a 4.0 ml centrifugation tube (Beckman Coulter, USA). Thereafter, 1.7 ml 35% sucrose was layered on top, followed by 0.85 ml 5% sucrose. The sucrose gradient was centrifuged in the ultracentrifuge (Optima XE-90 ultracentrifuge, Beckman Coulter, USA) for 20 h at 32,000 rpm (140,000 g) and 4 °C with slow deceleration. 9 DRM fractions were isolated (counting from the top of the sucrose gradient) at a volume of 450 μl with the second and third fraction containing the DRMs (shown by cholesterol and sphingolipid content). The pellet was resuspended in 450 µl MES-buffer and referred to as fraction 10. The samples were stored at −80 °C. Protein levels were determined using the Bicinchoninic acid (BCA) method.

### Real-time qPCR

mRNA from WBCs and from BMCs was extracted using the RNeasy Mini Kit (Qiagen, Hilden, Germany) or RNA Mini Kit (Bio & Sell, Feucht, Germany) according to the manufacturer’s instructions. The cDNA synthesis was performed using a First Strand cDNA-Synthesis kit (Thermo Scientific, Schwerte, Germany) including random hexamers. The expression levels of CXCR2, G-CSF-R, SOCS3 and peptidyl prolyl isomerase A (PPIA) were determined using Maxima SYBR Green (Thermo Scientific, Schwerte, Germany) with an ABI Prism 7500 Sequence Detection System (Applied Biosystems, Austin, USA). Relative mRNA expression was determined using the comparative CT (cycle threshold) method, normalizing relative values to the expression level of murine PPIA. The sequences for the primer sets of CXCR2 and PPIA are published^2^. The primer set for G-CSF-R are for 5′TGG CAA AAT GGT AGG GCT GG3′ and rev 5′GAT GCT GTC TGT CCC CAG GTT G3′ and for SOCS3 for 5′GCT CCA AAA GCG AGT ACC AGC3′ and rev 5′AGT AGA ATC CGC TCT CCT GCA G3′. Linearity of the assays was determined by serial dilutions of the templates for each primer set separately.

### Determination of sphingolipid levels

For quantification of sphingolipids, high-performance liquid chromatography/tandem mass spectrometry (LC-MS/MS) was used. 50 µl sample were mixed with 150 µl water, 150 µl extraction buffer (citric acid 30 mM, disodium hydrogen phosphate 40 mM) and 20 µl of the internal standard solution containing C17:0-Cer, C16:0-Cer-d31, C18:0 Cer-d3, C17:0-LacCer C16:0-LacCer-d3 (Avanti Polar Lipids, Alabaster, USA) and C24:0-Cer-d4 (Chiroblock GmbH, Bitterfeld-Wolfen) (400 ng/ml each). The mixture was extracted once with methanol/chloroform/hydrochloric acid (15:83:2, v/v/v). The organic phase was evaporated at 45 °C under a gentle stream of nitrogen and reconstituted in 200 µl of tetrahydrofuran/water (9:1, v/v) with 0.2% formic acid and 10 mM ammonium formate. Afterwards, 10 µL of the reconstituted solution were analyzed using a Zorbax EclipsePlus C18 column (50 mm × 2.1 mm ID, 1.8 μm particle size; Agilent technologies, Waldbronn, Germany) and water with 0.2% formic acid and 2 mM ammonium formate and acetonitrile/isopropanol/acetone (50:30:20, v/v/v) with 0.2% formic acid as mobile phases run in gradient elution mode. Total running time was 19.5 min. MS/MS analyses were performed using a mass spectrometer 5500QTRAP (Sciex, Darmstadt, Germany), operating as triple quadrupole, in positive electrospray ionization mode and Multiple Reaction Monitoring (MRM) mode.

Data Acquisition was done using Analyst Software V 1.6 and quantification was performed with MultiQuant Software V 3.0 (both Sciex, Darmstadt, Germany), employing the internal standard method (isotope dilution mass spectrometry). Calibration ranges were ranged from 0.05 to 5 ng/sample, 0.2 to 20 ng/sample and 2 to 200 ng/sample depending on the analyte.

### G-CSF-R and CXCR2 ELISA

The G-CSF-R ELISA kit was purchased from MyBioSource (San Diego, USA) and the CXCR2 ELISA kit was from Cloud-Clone Corp. (Houston, USA). The DRM fractions were diluted 1:10 in VE- H_2_0 and the concentrations of G-CSF-R and CXCR2 were determined according to the manufacturer’s instructions using an Infinite 200 PRO Reader (Tecan, Crailsheim).

### Preparation of protein extracts

BMCs of WT, CerS2 null and CerS6 null mice were incubated for 5, 10, 20 and 40 min with 10 ng/ml recombinant mouse G-CSF. Cell pellets were homogenized and sonicated in PhosphoSafe Extraction Reagent (Merck, Darmstadt, Germany) and supplemented with 1:10 Pefabloc (Sigma, Munich, Germany). The homogenate was centrifuged, the supernatants were collected and stored at −80 °C. Protein concentrations were assessed using the Bradford method.

### Western Blotting

Samples of 30–60 µg of total protein extract were separated electrophoretically by 8% SDS-PAGE and electroblotted onto nitrocellulose membranes (Amersham Life Science, Freiburg, Germany). Membranes were blocked in 5% bovine serum albumin (BSA) in PBS and incubated with the respective primary antibody directed against P-Src (detection of Src family tyrosine kinases Lyn/Fyn/Yes/Lck/Hck; 1:100), Lyn (1:100), P-STAT3 (1:100) and STAT3 (1:100). All antibodies were diluted in 5% BSA in 0.1% Tween 20 in PBS. Membranes were washed three times with 0.1% Tween 20 in PBS and then incubated with an IRDye800 or IRDye700 conjugated secondary antibody (BIOTREND Chemikalien GmbH, Köln, Germany) in Odyssey Blocking Buffer: 0.1% Tween 20 in PBS (1:1) for 2 h at RT. Membranes were analysed on the Odyssey infrared scanner from LI-COR (Bad Homburg, Germany). Anti-P-Src, anti-P-STAT3 and anti-STAT3 antibodies were purchased from Cell Signaling (Leiden, Netherlands) and anti-Lyn was purchased from Biolegend (Fell, Germany).

### FACS analysis

BMCs were treated with 10 ng/ml recombinant mouse G-CSF for 24 h and 46 h to check for CXCR2 surface expression on neutrophils. One hour before G-CSF treatment, the cells were incubated with 10 µM Lyn inhibitor PP1 (Tocris Bioscience, Bristol, UK) and then with an antibody cocktail consisting of CD11b-eFluor450 (eBioscience, Frankfurt, Germany), Ly6G-APC-Cy7 (BD, Heidelberg, Germany) and CXCR2-APC (BioLegend, Fell, Germany) for 15 min at room temperature. Samples were acquired with a MACSQuant Analyzer 10 flow cytometer (Miltenyi Biotec, Bergisch Gladbach, Germany) and analyzed using FlowJo software v10 (Treestar, Ashland, USA). All antibodies were previously titrated to determine optimal concentrations. Antibody-capturing CompBeads (BD, Heidelberg, Germany) were used for single-color compensation to create multi-color compensation matrices.

### Reporter gene assay-cloning of putative CerS2 constructs

The putative promoter region of murine CerS2 was assumed to lie upstream of exon 1, in exon 1 or in intron 1, because the ATG lies in exon 2 (Supplemental 1A). Various promotor constructs (prom2_I-V) from the putative promoter regions (Supplemental 1A) of CerS2 gene were amplified by PCR using genomic DNA from murine BMCs. Primers, used for the PCR, contained an identification restriction site (Table 1). PCR was performed with Advantage HD Polymerase (Clontech, Mountain View, USA). PCR conditions: 1 min 98 °C, 10 s 98 °C, 5 s 55 °C, 2 min 72 °C, repeated 34 times, ending with a final 72 °C step for 10 min. The resulting CerS2 promoter PCR products were subcloned in pCRII-TOPO, then digested with XhoI and SacI and cloned into the pGL3 Basic vector (Promega, Fitchburg, USA) and further designated as prom2_I-V.

**Table 1:** Primer for cloning strategy of CerS2-promoter constructs (prom2_I-V) and CerS6 promotor construct (prom6).

Primer	Sequence	cloning restriction sites	Construct size (kbp)
Prom2_I - for	TCTAAGTAAGCTTCAGACAGGCTGGAAATCATC	HindIII, KpnI/XhoI	1,657
Prom2_I - rev	GAATGCCAAGCTTTCCCTCCTTCCTCCTTTG	HindIII, KpnI/XhoI	1,657
Prom2_II - for	TCTAAGTAAGCTTGTTAGCCAGTGCCCAAAC	HindIII, EcoRI	1,977
Prom2_II - rev	GAATGCCAAGCTTACCCGCGCACAGACTTACTC	HindIII, EcoRI	1,977
Prom2_III - for	TATCCATGGGTACCAGAAGTGGGAAACGGAGTAG	KpnI	4,779
Prom2_III - rev	TATCCATGGGTACCAGCATCCTGAGTGAGGAAAG	KpnI	4,779
Prom2_IV - for	Restricted from Prom2_III	EcoRI	4,079
Prom2_IV - rev	Restricted from Prom2_III	EcoRI	4,079
Prom2_V - for	TATCCATGGGTACCAAATCATGGGCTGAGGTC	KpnI	1,180
Prom2_V - rev	TATCCATGGGTACCCCTCACAGCGCTTAGAAC	KpnI	1,180
Prom6 - for	TCTAAGTAAGCTTAGCCAAACAGGTAGTGGAGGGTG	HindIII	1,578
Prom6 - rev	GAATGCCAAGCTTGCTTTGTCCACTCCGGTG	HindIII	1,578

### Reporter gene assay-cloning of putative CerS6 constructs

The putative promoter region of the murine CerS6 was assumed to lie upstream of exon 1 or in exon 1, because the ATG lies in exon 1 (Supplemental 1B). One promotor construct (prom6) from the putative promoter region (Supplemental 1B) of the CerS6 gene was amplified by PCR using genomic DNA from murine BMCs. Primers used for the PCR contained an identification restriction site (Table 1). PCR was performed with Advantage HD Polymerase (Clontech, Mountain View, USA). PCR conditions: 2 min 94 °C, 15 s 94 °C, 20 s 59 °C, 1 min 40 s 72 °C, repeated 34 times, ending with a final 72 °C step for 7 min. The resulting CerS6 promoter PCR products were digested with HindIII and cloned into the pGL3 Basic vector (Promega, Fitchburg, USA) and further designated as prom6.

### Reporter gene assay

Transient transfection was performed with MACSfectin (Miltenyi Biotec, Bergisch Gladbach, Germany) according to the manufacturer’s protocol. 1.2 × 10^5^ RAW247 macrophage cells were seeded into 96-well plates one day before transfection. The cells were cultured three days before and during the experiment in RPMI 1640 medium – GlutaMAX containing 2.5% charcoal stripped FCS for putative CerS2 promotor constructs and 2.5% normal FCS for CerS6 promotor constructs. The cells were transfected with 125 ng of the distinct firefly luciferase reporter vector (pGL3 Basic vector, pGL3 prom2_I-V or prom6 vector) and 12.5 ng of the Renilla luciferase control reporter vector (pRL-TK, Promega, Fitchburg, USA) using 0.5 μl MACSfectin reagent. One day after transfection, the cells were incubated for 46 h with or without 10 ng/ml G-CSF. The luciferase assay was performed with luciferase substrate buffer (20 mM tricine; 2.67 mM magnesium carbonate ((MgCO_3_)_4_Mg(OH)_2_); 1.07 mM MgSO_4_; 100 μM ethylenediamine tetraacetic acid (EDTA); 0.33 mg/ml 3.3′-diethylthiatricarbocyanine iodide (DTTC); 0.21 mg/ml co-enzyme A; 470 mM d-luciferin) in a luminometer (Infinite F200 Pro, Tecan, Crailsheim, Germany) according to the manufacturer’s instructions. The activity of firefly luciferase was normalized to the RLU of renilla luciferase (renilla substrate buffer: 0.1 M NaCl; 25 mM Tris–HCl [pH 7.5]; 0.147 mg/ml CaCl_2_; 900 nM Coelenterazine).

### Statistics

Results are presented as means ± SEM (standard error of the mean) or ± SD (standard deviation). Significant differences in multiple data sets were analyzed by Two-way ANOVA with Bonferroni post hoc-test or alternatively with Tukey’s or Sidak’s multiple comparisons test. Significant differences between two groups were analyzed by t-test (Graphpad Prism 6 software). The level of significance was set at p < 0.05.

## Supplementary information

Supplemental Information

## Data Availability

All data generated or analysed during this study are included in this published article (and its Supplementary Information files).
