# Supplementary material for: Role of ceramide synthase 2 in G-CSF signaling and G-CSF-R translocation into detergent-resistant membranes
Source: Sci Rep. 2019 Jan 24;9:747. doi: 10.1038/s41598-018-37342-8 (PMC6345911; doi:10.1038/s41598-018-37342-8)
Supplement: Supplementary file 1 — Supplemental Information [file 41598_2018_37342_MOESM1_ESM.pdf]

Role of ceramide synthase 2 in G-CSF signaling and G-CSF-R translocation into detergent-resistant membranes

Running title: Role of ceramide synthase 2 in G-CSF signaling

Jennifer Kurz[1], Julia Barthelmes[2], Leonard Blum[2], Thomas Ulshöfer[1], Marthe-Susanna Wegner[2], Nerea Ferreirós[2], Luise Roser[1], Gerd Geisslinger[2], Sabine Grösch[† 2] and Susanne Schiffmann[†*1]

[1]Fraunhofer Institute for Molecular Biology and Applied Ecology IME, Branch for Translational Medicine and Pharmacology TMP, Theodor-Stern-Kai 7, 60596 Frankfurt am Main

[2]*pharmazentrum frankfurt/ZAFES,* Institute of Clinical Pharmacology, Goethe University Hospital Frankfurt, Theodor-Stern-Kai 7, 60590 Frankfurt/Main, Germany

[†] Authors contributed equally

***Corresponding author:** Susanne Schiffmann, PhD; Institute of Clinical Pharmacology;
Fraunhofer IME-TMP; Industriepark Höchst, Bd G879; 65926 Frankfurt/Main, Germany
Phone +49 69-8700-25060; Fax +49 69-8700-10000; susanne.schiffmann@ime.fraunhofer.de

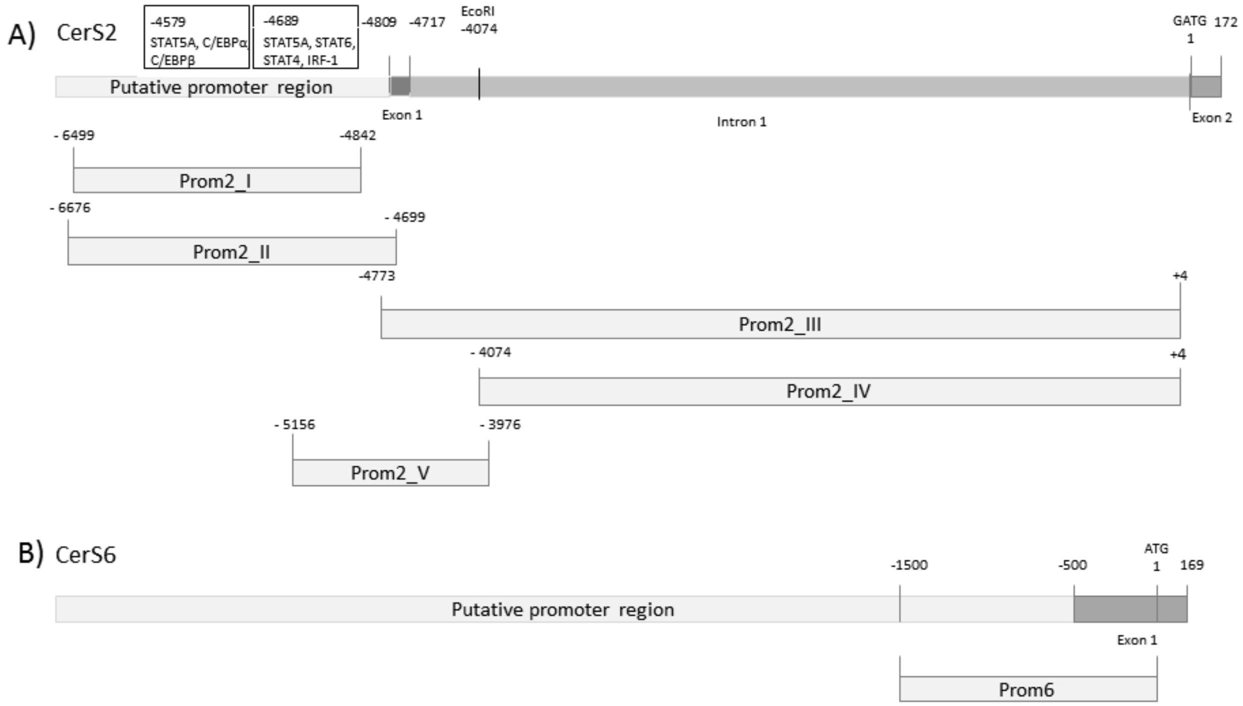

Supplemental 1: Cloning strategy for CerS2 (A) and CerS6 (B) promoter constructs. A) Promotor construct 1 (Prom2_I) compromises 1657 Bp from the upstream region, construct 2 (prom2_II) includes additionally a part of 110 Bp of exon 1. Construct 3 (prom2_III) comprises 56 Bp of exon 1 and intron 1. Construct 4 (prom2_IV) comprises 4074 Bp of intron 1. Construct 5 (prom2_V) comprise 347 Bp of the upstream region, exon1 and 741 Bp from intron1. The sequence from NM_029789.1 was used and the first Base of exon 2 was assigned as 1. Putative transcription binding factor sites are added for the putative promotor region, including STAT5A, C/EBPα, C/EBPβ, STAT6, STAT4 and IRF-1. B) The promotor construct for CerS6 (prom6) comprises 1000 Bp from the upstream region and 500 Bp from exon 1.The sequence from NM_172856.3 was used and A of ATG in Exon 1 was assigned as 1.

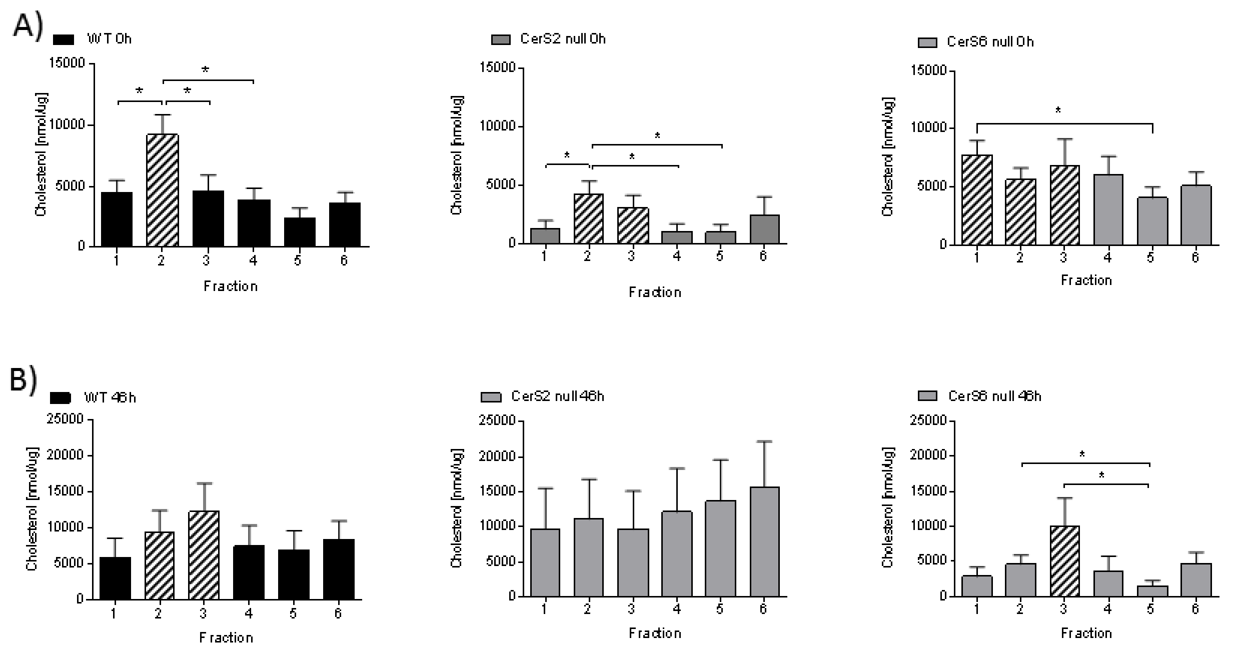

Supplemental 2: Cholesterol concentrations in WT, CerS2 null and CerS6 null bone marrow cells before (0 h) and after treatment with G-CSF (46 h). Cell lysates were separated by succrose gradient centrifugation and divided into 10 fractions. From each fraction the cholesterol concentration was determined by colorimetric cholesterol assay (absorbance 540 nm). 10 µl standards dissolved in 100 % ethanol and 10-50 µl samples were added to 5 µl of emulsifier solution (15 % Brij in 5 mM Tris/HCl pH 7.5) and incubated in a 96 well plate at RT for 15 min. Afterwards 200 µl of the reaction solution was added and the plate was incubated for 60 min at 37 °C. The reaction solution was freshly prepared at a ratio of 1:1:0.01 of solution A (0.02 M phenol, 1.84 M MeOH, 1 % methyl-β-cyclodextrin, ad 50 mM Tris/HCl pH 7.4), solution B (0.0019 M 4-aminoantipyrin, 1.84 M MeOH, 0.4 % Brij (30 % w/v), ad 50 mM Tris/HCl pH 7.4 and enzyme solution. The enzyme solution consisted of cholesterol oxidase (12 U/ml) and peroxidase (8 U/ml). The figures represent 3 (CerS2 null, CerS6 null) - 11 (WT) independent experiments. For each experiment the bone marrow cells of two mice were pooled. Dashed bars indicate DRM fractions. Data are mean ± SEM. * ($p < 0.05$).

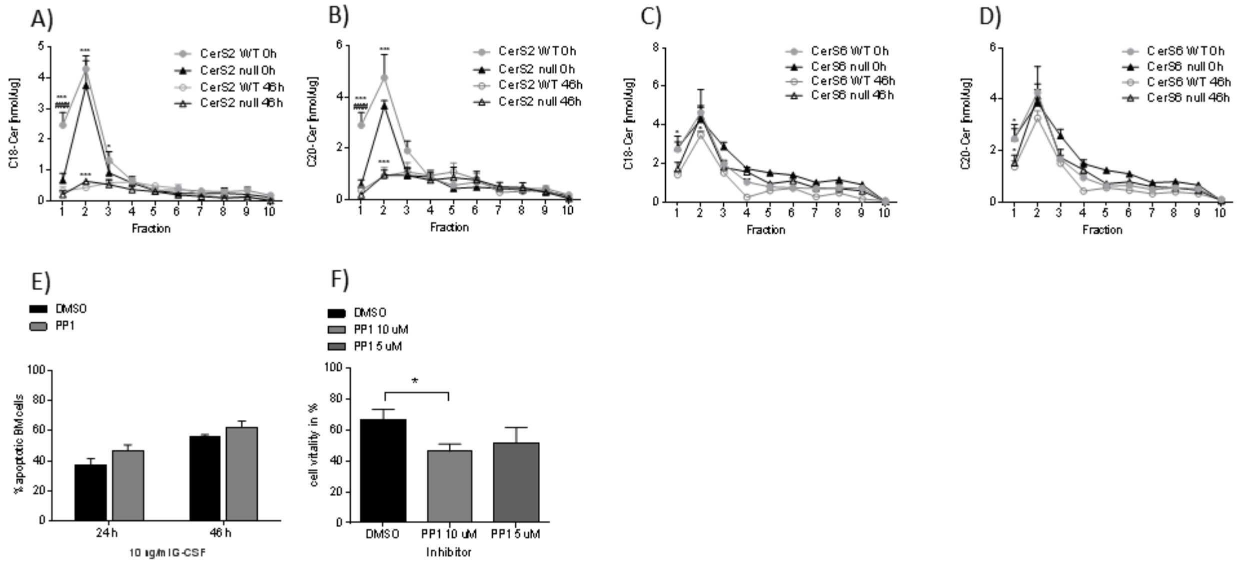

Supplemental 3: A-D) Ceramide levels in WT, CerS2 null and CerS6 null bone marrow cells before (0 h) and after treatment with G-CSF (46 h). Cell lysates were separated by succrose gradient centrifugation and divided in 10 fractions. From each fraction the ceramide levels were determined by LC-MS/MS. The figures represent 3 independent experiments. For each experiment the bone marrow cells of two mice were pooled. LC-MS/MS detection range: C18-Cer (400-20.000 pg/sample), C20-Cer (50-5.000 pg/sample). E) Apoptosis rate in WT bone marrow cells treated with 10 ng/ml G-CSF for 24 h and 46 h in presence of 10 µM PP1 (Lyn inhibitor) or DMSO (control). Apoptosis was determined using Annexin/7-AAD staining by flow cytometry. The figure represents two independent expemeriments. Each experiment was achieved in triplicate. F) Cell viablity in RAW cells transfected with pGL3 and treated with 5 µM and 10 µm PP1 (Lyn inhibitor) compared to DMSO control. Cell viability was determined by WST assay. The figure represents 4-6 independent experiments. Each experiment was achieved in quadruple. Data are mean ± SEM. * ($p < 0.05$) ** ($p < 0.01$), *** ($p < 0.001$) indicates significant differences between time point 0 h and 46 h as well as between DMSO and PP1 treatment (A-D: Two-way ANOVA; F: t-test). # ($p < 0.05$) ## ($p < 0.01$), ### ($p < 0.001$) indicates significant differences between WT and CerS2 null or CerS6 null cells at a specific time point (Two-way ANOVA).

Supplemental 4: Statistics of ceramide content in different DRM fractions. Analysis of 5 DRM fractions (F1-F5) to identify the fractions with the highest ceramide content. DRM samples from CerS2/6 WT and null BMCs were investigated for C16-Cer, C18-Cer, C20-Cer, C24-Cer, C16-LacCer and C24-LacCer by LC-MS/MS. ns = not significant, n.d.= not detectable, + $p<0.05$, ++ $p< 0.01$, +++ $p< 0.001$ fractions were analysed against each other (F:F) by Two-way ANOVA.

	**F1:F2**	**F1:F3**	**F1:F4**	**F1:F5**	**F2:F3**	**F2:F4**	**F2:F5**	**F3:F4**	**F3:F5**	**Fraction(s) with highest ceramide content**
CerS2 WT 0 h **C16**	+	ns	+	++	+++	+++	+++	ns	ns	F2
CerS2 null 0 h **C16**	+++	ns	ns	ns	+++	+++	+++	ns	ns	F2
CerS2 WT 0 h **C18**	+++	ns	+++	+++	+++	+++	+++	ns	ns	F1, F2
CerS2 null 0 h **C18**	+++	ns	ns	ns	+++	+++	+++	ns	ns	F2
CerS2 WT 0 h **C20**	+++	ns	+++	+++	+++	+++	+++	ns	+	F1, F2
CerS2 null 0 h **C20**	+++	ns	ns	ns	+++	+++	+++	ns	ns	F2
CerS2 WT 0 h **C24**	+++	ns	+++	+++	+++	+++	+++	ns	+	F2
CerS2 null 0 h **C24**	ns	ns	ns	ns	ns	ns	ns	ns	ns	
CerS2 WT 0 h **LacCer16**	ns	ns	ns	ns	ns	ns	ns	ns	ns	
CerS2 null 0 h **LacCer16**	+++	ns	ns	ns	+++	+++	+++	ns	ns	F2
CerS2 WT 0 h **LacCer24**	ns	ns	ns	ns	ns	+	+++	ns	ns	F2
CerS2 null 0 h **LacCer24**	ns	ns	ns	ns	ns	ns	ns	ns	ns	

	F1:F2	F1:F3	F1:F4	F1:F5	F2:F3	F2:F4	F2:F5	F3:F4	F3:F5	Fraction(s) with highest ceramide content
CerS2 WT 46 h **C16**	ns	ns	ns	ns	ns	ns	ns	ns	ns	
CerS2 null 46 h **C16**	+	ns	ns	ns	ns	ns	ns	ns	ns	
CerS2 WT 46 h **C18**	ns	ns	ns	ns	ns	ns	ns	ns	ns	
CerS2 null 46 h **C18**	+	ns	ns	ns	ns	ns	ns	ns	ns	
CerS2 WT 46 h **C20**	ns	ns	ns	ns	ns	ns	ns	ns	ns	
CerS2 null 46 h **C20**	ns	ns	ns	ns	ns	ns	ns	ns	ns	
CerS2 WT 46 h **C24**	ns	++	+	ns	ns	ns	ns	ns	ns	
CerS2 null 46 h **C24**	ns	ns	ns	ns	ns	ns	ns	ns	ns	
CerS2 WT 46 h **LacCer16**	ns	ns	ns	ns	ns	ns	ns	ns	ns	
CerS2 null 46 h **LacCer16**	+	ns	ns	ns	ns	+	+	ns	ns	
CerS2 WT 46 h **LacCer24**	ns	ns	ns	ns	ns	ns	ns	ns	ns	
CerS2 null 46 h **LacCer24**	ns	ns	ns	ns	ns	ns	ns	ns	ns	

	F1:F2	F1:F3	F1:F4	F1:F5	F2:F3	F2:F4	F2:F5	F3:F4	F3:F5	Fraction(s) with highest ceramide content
CerS6 WT 0 h **C16**	+++	ns	+++	+++	+++	+++	+++	+	++	F2
CerS6 null 0 h **C16**	ns	ns	ns	ns	ns	ns	ns	ns	ns	
CerS6 WT 0 h **C18**	+++	ns	+++	+++	+++	+++	+++	ns	ns	F1, F2
CerS6 null 0 h **C18**	++	ns	ns	++	++	+++	+++	+	++	F1, F2
CerS6 WT 0 h **C20**	+++	ns	+++	+++	+++	+++	+++	ns	ns	F1, F2
CerS6 null 0 h **C20**	++	ns	+	++	++	+++	+++	+	++	F2
CerS6 WT 0 h **C24**	+++	ns	+++	+++	+++	+++	+++	+	++	F1, F2
CerS6 null 0 h **C24**	+	ns	ns	+	ns	+++	+++	ns	++	F2
CerS6 WT 0 h **LacCer16**	n.d.	n.d.	n.d.	n.d.	n.d.	n.d.	n.d.	n.d.	n.d.	
CerS6 null 0 h **LacCer16**	n.d.	n.d.	n.d.	n.d.	n.d.	n.d.	n.d.	n.d.	n.d.	
CerS6 WT 0 h **LacCer24**	ns	ns	n.d.	n.d.	ns	n.d.	n.d.	n.d.	n.d.	
CerS6 null 0 h **LacCer24**	ns	ns	n.d.	n.d.	ns	n.d.	n.d.	n.d.	n.d.	

	**F1:F2**	**F1:F3**	**F1:F4**	**F1:F5**	**F2:F3**	**F2:F4**	**F2:F5**	**F3:F4**	**F3:F5**	**Fraction(s) with highest ceramide content**
CerS6 WT 46 h **C16**	+++	ns	ns	ns	+++	+++	+++	+++	++	F2
CerS6 null 46 h **C16**	+	ns	ns	ns	+	++	++	ns	ns	F2
CerS6 WT 46 h **C18**	++	ns	ns	ns	++	+++	+++	ns	ns	F2
CerS6 null 46 h **C18**	+++	ns	ns	ns	+++	+++	+++	ns	ns	F2
CerS6 WT 46 h **C20**	+++	ns	ns	ns	++	+++	+++	ns	ns	F2
CerS6 null 46 h **C20**	+++	ns	ns	ns	+++	+++	+++	ns	ns	F2
CerS6 WT 46 h **C24**	+++	ns	++	++	+++	+++	+++	++	++	F1, F2, F3
CerS6 null 46 h **C24**	+++	ns	ns	ns	+++	+++	+++	ns	ns	F2
CerS6 WT 46 h **LacCer16**	+++	ns	n.d.	n.d.	+++	n.d.	n.d.	n.d.	n.d.	F2
CerS6 null 46 h **LacCer16**	ns	ns	n.d.	n.d.	ns	n.d.	n.d.	n.d.	n.d.	
CerS6 WT 46 h **LacCer24**	+++	ns	ns	ns	+++	+++	+++	ns	ns	F2
CerS6 null 46 h **LacCer24**	+++	ns	ns	ns	+++	+++	+++	ns	++	F2

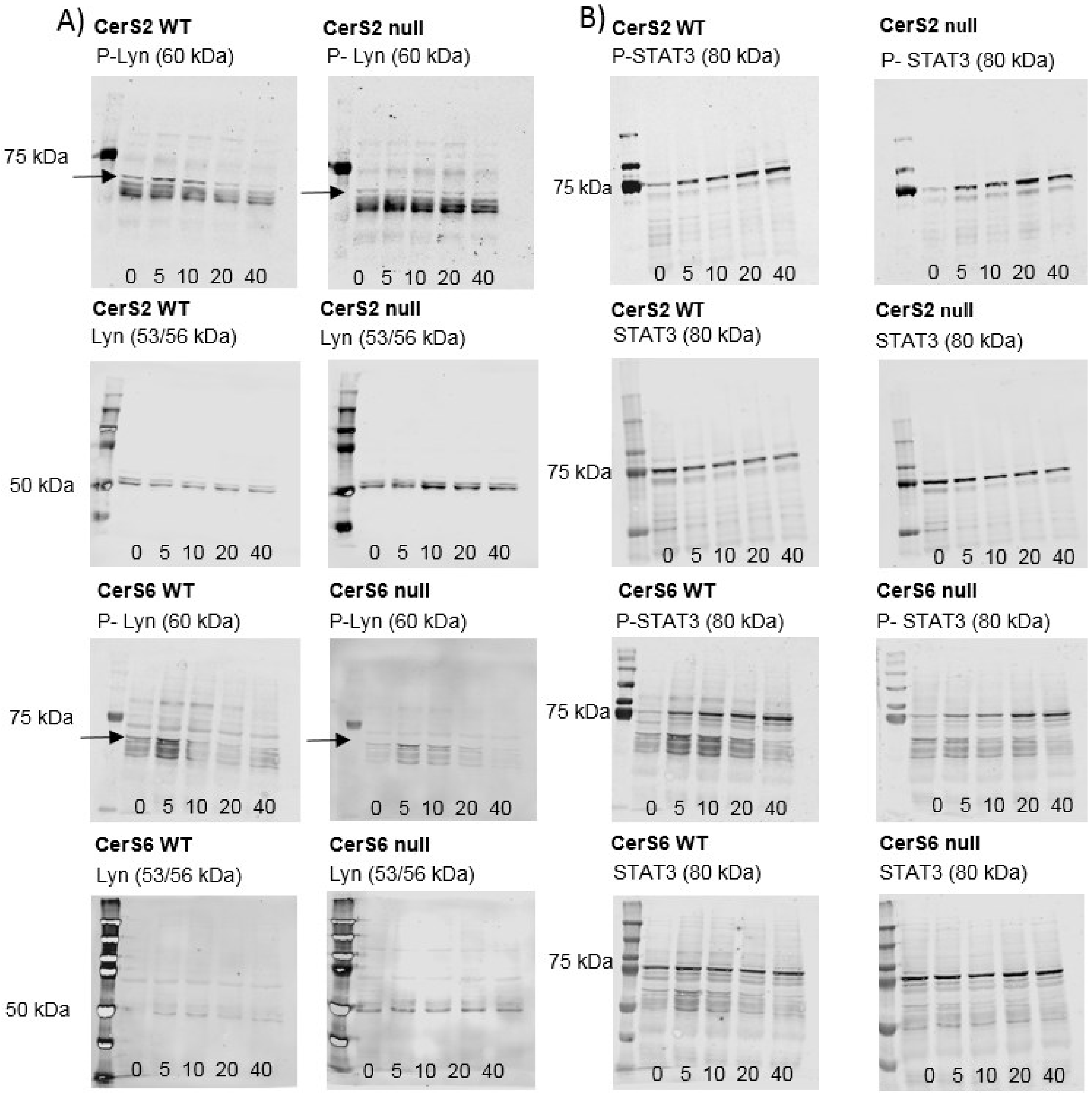

Supplemental 5: Downstream signaling of G-CSF is regulated in a CerS2- and CerS6-dependent manner. A) Full-length western blot of P-Lyn kinase (60 kDa) and Lyn kinase (53/56 kDa) B) Full-length western blot of P-STAT3 (80 kDa) and STAT3 (80 kDa). Bone marrow cells (BMCs) from WT, CerS2 null and CerS6 null mice were incubated for different times with 10 ng/ml G-CSF (0, 5, 10, 20 and 40 min) and the phosphorylation of Lyn kinase and STAT3 was determined using western blot. The relative phosphorylation status of the kinases and the transcription factor were calculated using the unphosphorylated protein as reference. A representative western blot of three is shown.
